# Exercise-Induced Improvements in Postprandial Glucose Response Are Blunted by Pre-Exercise Hyperglycemia: A Randomized Crossover Trial in Healthy Individuals

**DOI:** 10.3389/fendo.2020.566548

**Published:** 2020-10-15

**Authors:** Steven Carter, Thomas P. J. Solomon

**Affiliations:** ^1^School of Sport, Exercise, and Rehabilitation Sciences, College of Life and Environmental Sciences, University of Birmingham, Edgbaston, United Kingdom; ^2^Institute of Systems and Metabolism Research, College of Medical and Dental Sciences, University of Birmingham, Edgbaston, United Kingdom

**Keywords:** exercise, type 2 diabetes, postprandial, glycemic control, hyperglycemia, heterogeneity, variability

## Abstract

**Background:**

Exercise improves glycemic control but the magnitude, and in some cases, the direction of this effect is variable. Ambient hyperglycemia has been implicated in this exercise response heterogeneity. The current study investigated whether pre-exercise hyperglycemia directly impacts the effect of exercise on glycemic control.

**Methods:**

Twelve healthy normal glucose-tolerant males completed four trials in a randomized, crossover design. Each trial consisted of 24-h pre-intervention monitoring, a 7-h intervention, and 24-h post-intervention monitoring. Glycemic control was measured throughout the study by continuous glucose monitoring. The four interventions were no exercise (CON) or 45 min of cycling exercise (70%HRmax) preceded by 3.5 h of either normoglycemia (NG-Ex), steady-state hyperglycemia induced by constant glucose infusion (HG-Ex) or fluctuating glycemia induced by repeated glucose bolus infusions (FG-Ex).

**Results:**

Physical activity and diet were similar between trials, and energy expenditure during exercise was matched between exercise trials (all *P* > 0.05). Mean glucose during the 3.5 h ± infusion period was higher in HG-Ex (mean ± SEM; 7.2 ± 0.4 mmol/L) and FG-Ex (7.3 ± 0.3 mmol/L) compared to CON (4.8 ± 0.2 mmol/L) and NG-Ex (5.0 ± 0.2 mmol/L) trials (*P* < 0.01). Glycemic variability was greatest in FG-Ex (*P* < 0.01). Following the interventions, the postprandial glucose response (iAUC) was reduced by exercise in NG-Ex compared to CON (321.1 ± 38.6 vs. 445.5 ± 49.7 mmol/L.8h, *P* < 0.05, *d*=0.81). This benefit was blunted when exercise was preceded by steady-state (HG-Ex, 425.3 ± 45.7 mmol/L.8h) and fluctuating (FG-Ex, 465.5 ± 39.3 mmol/L.8h) hyperglycemia (both *P* > 0.05 vs. CON).

**Conclusion:**

Pre-exercise hyperglycemia blunted the glucoregulatory benefits of acute exercise upon postprandial glucose response, suggesting that exposure to hyperglycemia contributes to exercise response heterogeneity.

**Clinical Trial Registration:**

ClinicalTrials.gov, identifier NCT03284216.

## Introduction

The worldwide prevalence of type 2 diabetes mellitus (T2DM) continues to rise, meaning increasing proportions of the global population are at risk of or living with a range of serious microvascular and macrovascular complications (1–3). Consequently, the economic cost of managing T2DM continues to rise and places an increasing burden on healthcare systems. Optimizing interventions to improve glycemic control remains a clinical necessity.

The level of glycemia, and particularly postprandial glucose exposure (4–6), has been implicated with the aforementioned diabetic complications, as well as being predictive of a worsening glycated haemoglobin (HbA1c) level in non-diabetic individuals (7). Therefore, reducing postprandial glucose exposure is particularly important for the prevention and long-term management of glycemic control in individuals with or at risk of T2DM. Exercise training exerts potent glucoregulatory effects in those with and at risk of developing T2DM. For example, exercise training reduces HbA1c and fasting blood glucose (8, 9) as well as increases peripheral insulin sensitivity (10–12) and β-cell insulin secretory function (13, 14). A single exercise bout also potently increases glucose uptake, insulin sensitivity (15) and β-cell insulin secretory function (16) in the hours to days following each exercise bout. Similarly, and of particular relevance to the current study, reduced (i.e., improved) postprandial glucose response is also among the glucoregulatory effects of a single exercise bout (17–21). Many of these benefits are gained by individuals with and without T2DM, but the transient nature of benefits means exercise must be repeated regularly to preserve metabolic health. Accordingly, regular exercise forms the cornerstone in the prevention and management of hyperglycemia-related conditions, including T2DM (22).

While the potent glucoregulatory effects of exercise are unequivocal, the magnitude and direction of change following both acute exercise (16, 23, 24) and exercise training (25, 26) in hyperglycemic individuals vary considerably. Isolating factors contributing to this exercise response heterogeneity is vital to optimizing the glucoregulatory effects of exercise in this population and have been discussed in several recent reviews (27–30). In free-living environments, factors such as diet and exercise characteristics/adherence and exercise-medication interactions (31, 32) likely contribute to variability in this setting (30). Interestingly, evidence from recent well-controlled studies, where free-living sources of heterogeneity (e.g., exercise-drug interaction, exercise adherence, diet) are controlled, implicates the degree of hyperglycemia as one possible contributor to heterogeneity following single exercise bouts as well as exercise training (27, 28, 30). That said, equivocal conclusions have been made in studies to date, with both blunted (16, 33–35) and potentiated (23, 24) exercise-induced glucoregulatory benefits associated with higher baseline fasting plasma glucose and/or HbA1c. Furthermore, the evidence to date is also largely correlational, meaning that the direct effect of hyperglycemia on exercise-mediated improvements in glycemic control remains to be tested experimentally. Accordingly, the current study investigated the impact of pre-exercise hyperglycemia on the response to a single exercise bout in healthy normal glucose-tolerant participants. It was hypothesized that pre-exercise hyperglycemia would blunt the glucoregulatory effects of acute exercise.

## Materials and Methods

### Participants

Healthy, recreationally active males with normal glucose tolerance (*n* = 12; **Table 1**) were recruited from the local community to participate in the current study. Potential participants underwent an initial screening visit to determine their eligibility for the study. Individuals were excluded from participation if they smoked, had a BMI >30 kg/m^2^, and/or had a history of cancer, haematological, pulmonary, cardiac, hepatic, renal, metabolic, or gastrointestinal diseases. All participants provided written informed consent before participation. The CONSORT diagram in **Supplemental Figure S1** shows an overview of the recruitment/screening/inclusion decisions. Formal sample size calculations were made using G*Power Version 3.1.7 (36). Ethical approval was obtained through the West Midlands - South Birmingham Research Ethics Committee (16/WM/0242) and sponsored by the University of Birmingham Research Governance. The study is registered at ClinicalTrials.gov (NCT03284216).

**Table 1:** Participant characteristics.

N	12
Age, years	23.6 ± 1.5
Height, cm	175.1 ± 1.7
Weight, kg	69.5 ± 2.3
BMI, kg/m^2^	22.7 ± 0.7
Waist circumference, cm	76.5 ± 1.5
HbA1c, %	5.4 ± 0.1
HbA1c, mmol/mol	35.1 ± 1.0
Fasting plasma glucose, mmol/L	4.9 ± 0.2
HOMA-IR	1.1 ± 0.1
Systolic blood pressure, mmHg	121 ± 3
Diastolic blood pressure, mmHg	76 ± 3
V̇O_2_max, ml/kg/min	41.5 ± 3.3
V̇O_2_max, L/min	2.8 ± 0.2
W_max_, W	267.8 ± 13.9
W_max_, W/kg	3.8 ± 0.4
Habitual step count, steps/day	11452 ± 1578
Habitual energy expenditure, kcals/day	635.1 ± 103.0
Habitual sedentary time, min/day	726.9 ± 47.2
Habitual MVPA time, min/day	72.1 ± 11.4

Data are mean ± SEM.

### Screening and Habitual Monitoring

All participants completed a general health questionnaire, as well as had their body composition (height, weight, BMI, waist circumference) and HbA1c analyzed to confirm eligibility for the current study. Subsequently, participants performed a maximal incremental exercise test on a cycle ergometer (Lode Excalibur, Groningen, The Netherlands) to determine their maximum workload capacity (*W*max), maximal oxygen consumption (V̇O_2_max), and maximal heart rate (HRmax). After 5-min warm-up at 50 W, the workload was increased by 25 W/min until exhaustion. Oxygen consumption was assessed continuously during exercise *via* indirect calorimetry (Vyntus CPX, Jaeger, CareFusion, Germany), with heart rate (Polar Wearlink, Polar Electro Oy, Kempele, Finland), and ratings of perceived exertion (RPE; 6–20 Borg scale) monitored throughout. Following screening, participants completed 7 days of habitual monitoring, during which accelerometry (Actigraph wGT3X-BT, Pensacola, FL) and diet logs were used to assess habitual physical activity and diet, respectively.

### Study Design

**Figure 1:**
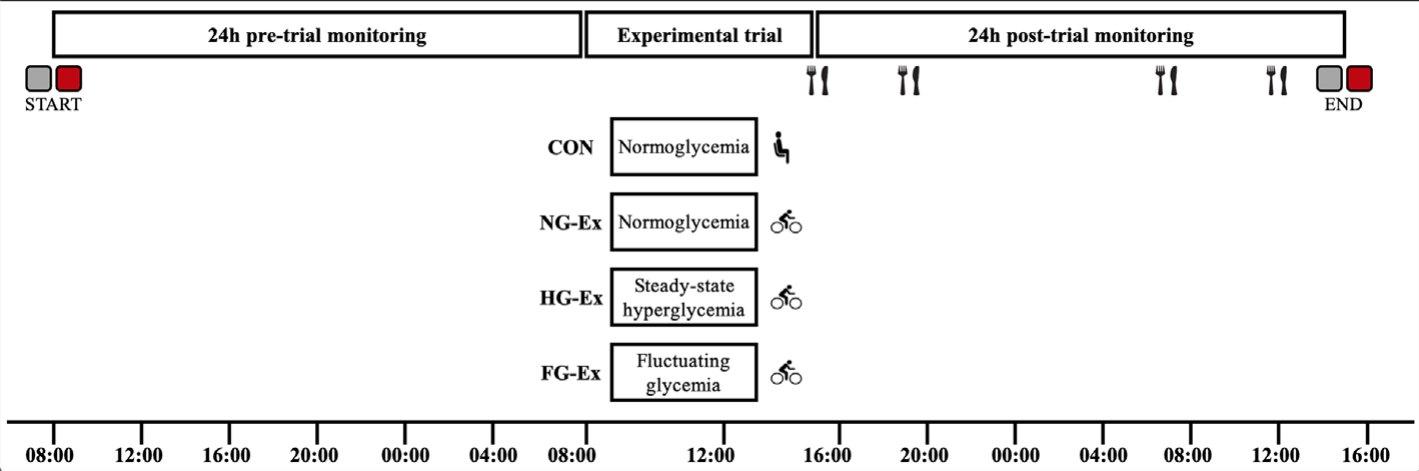
Schematic overview of the study design. Pre-trial monitoring involving physical activity (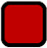) and CGM-derived glycemic control (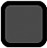) began 24 h before each experimental trial. During experimental trials, participants were exposed to differing glycemic profiles followed by 45 min of rest (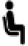) or exercise (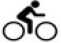). Post-trial glycemic control was assessed under strict dietary control (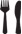) but otherwise free-living conditions. A full description is provided in the text.

Participants completed four experimental trials in a randomized, crossover design with trials separated by ~1 week, during which time participants were instructed to maintain habitual physical activity and diet. Each trial consisted of a 24-h pre-trial monitoring period, a 7-h experimental intervention period, and a 24-h post-trial monitoring period (**Figure 1**). The four 7-h interventions were completed in a randomized order (determined using http://www.randomization.com/) and were: normoglycemia, no exercise (CON), normoglycemia plus exercise (NG-Ex), steady-state hyperglycemia plus exercise (HG-Ex), and fluctuating glycemia plus exercise (FG-Ex).

### Pre-Trial Standardization and Monitoring Period

In the afternoon of day 0, participants reported to the laboratory for insertion of the continuous glucose monitoring (CGM) glucose sensor (Dexcom G5 mobile, Camberley, UK) into the abdominal subcutaneous adipose tissue, and to receive an accelerometer and diet records. Data was not collected on this day; instead, the purpose of this visit was to allow time for CGM calibration (which required ~2–3 h) in preparation for data collection to commence on day 1. The CGM was calibrated twice daily using a glucose meter (Contour Next One, Ascensia Diabetes Care UK, Berkshire, UK) on fingertip capillary blood samples. Participants were provided with detailed written instructions, including refraining from strenuous physical activity and the consumption of alcohol and caffeine from day 0 to day 3, inclusive.

On day 1 (24-h pre-trial), diet record (food type, amount and time of ingestion), accelerometry-derived physical activity, and CGM data collection commenced and continued until 24-h post-exercise (i.e., days 1–3, inclusive). Participants consumed a self-selected diet before trial 1 and then replicated this chosen diet on day 1 of subsequent trials. Similarly, participants were instructed to replicate as closely as possible activity patterns (e.g., mode of transportation) during the 24 h before each trial. This approach to pre-trial standardization is similar to other studies assessing the acute effects of exercise on glucose metabolism (37–39). Repeated verbal and written reminders of the importance of adhering to these standardizations were provided throughout.

### Experimental Trials

On day 2, participants reported to the laboratory at 08:00, after an overnight fast (≥10 h, except water). Bodyweight, height, and waist circumference were measured by standard procedures, and bilateral antecubital venous lines were placed for glucose infusion and blood sampling. The four trials were identical except for the pre-exercise glycemic intervention and the rest vs. exercise conditions. Specifically, after baseline measurements, one of four 3.5-h pre-exercise glycemic interventions commenced: CON (no infusion); NG-Ex (no infusion); HG-Ex, a constant glucose infusion of 1.2 g/kg for 3.5 h (5.71 mg/kg/min); and FG-Ex, a total of 1.2 g/kg of glucose infused over 3.5 h but divided into 8 equal boluses every 30 min (0.15 g/kg per bolus, infused over 3.5 min at 42.86 mg/kg/min). Glucose (20% w/v glucose monohydrate; Baxter FKB0213B) infusion was administered using a volumetric infusion system (Infusomat Space pump, B. Braun Medical Ltd, Sheffield, UK). Heart rate, blood pressure, oxygen saturation, and body temperature were measured throughout to monitor participant safety and well-being; no within- or between-trial differences were found (data not shown). Sixty minutes after cessation of the respective glycemic intervention (to allow for normalization of glucose levels), participants completed either a 45-min rest period (CON; remain seated) or a 45-min exercise bout (NG-Ex, HG-Ex, and FG-Ex; stationary cycling at 70% HRmax). Indirect calorimetry, heart rate and power output were measured continuously throughout the exercise/rest period. The end of the 45-min ± exercise period marked the start of the 24-h post-trial monitoring period.

### Post-Trial Standardization and Monitoring Period

For the remainder of day 2 until ~15:00 on day 3 (i.e., the 24-h post-exercise period), CGM, accelerometry, and diet record monitoring continued in free-living conditions. During this time, participants were instructed to minimise any unnecessary physical activity (e.g., walking around at home was permitted but structured exercise was not) and to eat only the standardized meals provided.

The standardized 24-h diet consisted of four meals ingested at predetermined time points (lunch at 15:00, dinner at 19:00, breakfast at 07:00, lunch at 12:00). The first meal (lunch) was consumed after 30-min seated rest following the cessation of the ± exercise period, after which participants were free to leave the laboratory. The standardized 24-h diet provided 2467.6 ± 4.6 kcals and consisted of a mixed macronutrient composition (54.9 ± 0.1% of energy from carbohydrates, 31.6 ± 0.1% from fat, and 13.5 ± 0.1% from protein). Participants were instructed to consume meals within a 20-min timeframe and recorded the exact meal ingestion start and end times to allow specific data to be extracted from CGM data files. This approach ensured that the dietary intake (food type, amount and timing) was standardized within-subjects across all four trials; an important approach frequently adopted when using CGM to evaluate exercise-induced changes in measures of glycemic control in free-living settings (20, 21, 38, 40–43). In the afternoon of day 3 (≥24 h following exercise cessation), participants reported to the laboratory for removal of CGM, accelerometer and collection of diet records and activity logs, marking the end of the trial.

It should be noted that no additional calories were provided to replace those expended during exercise in the exercise trials (NG-Ex, HG-Ex, FG-Ex) compared to CON (**Table 3**), or to account for extra calories gained from glucose infusions in HG-Ex and FG-Ex (83.5 ± 2.8 g of glucose infused, equating to 333.8 ± 11.2 kcals). Resultant energy balance induced by experimental interventions (i.e., crudely calculated as kcal gained during infusion minus kcal expended during exercise) is as follows: CON = 61.7 ± 3.6 kcals deficit; NG-Ex = 326.1 ± 18.8 kcals deficit; HG-Ex = 10.9 ± 20.5 kcals surplus; FG-Ex = 9.8 ± 17.7 kcals surplus). Although caloric imbalances between trials may be a confounder (44), accounting for imbalances between trials with the provision of food, for example, would result in differences in macronutrient provision between trials, which in itself would also be a confounding variable. Therefore, the meals were kept constant across all conditions.

### Blood Sample Analyses

HbA1c was measured in capillary blood samples during the screening visit (HemoCue HbA1c 501, Radiometer, Copenhagen, Denmark). Venous blood samples were collected in pre-chilled EDTA-coated tubes and centrifuged at 2,000 g for 15 min at 4°C, with resulting plasma samples stored at −80°C until analysis. Plasma insulin (DINS00), plasma IL-6 (HS600C), and plasma CRP (DCRP00) concentrations were determined using solid-phase sandwich ELISAs (Quantikine, Biotechne, R&D). Plasma glucose concentrations were measured using a HemoCue Glucose 201+ analyzer (Radiometer, Copenhagen, Denmark).

### Calculations

Diet records were analyzed using the UK food database within MyFitnessPal (Under Armour, Baltimore, MD). The 24-h pre-trial glucose and physical activity data were derived from CGM and Actigraph data, respectively, from 08:00 on the day before the trial to 08:00 on the day of the trial. The 24-h post-trial glucose and physical activity data were derived from CGM and Actigraph data, respectively, from the end of exercise until 24-h later. CGM-derived measures of glucose exposure (mean glucose and incremental area under the curve, iAUC) and glycemic variability (standard deviation and coefficient of variation, CV%) were calculated following clinical guidelines (45). The 24-h prevalence of hyperglycemia (time spent above 8 mmol/L) and hypoglycemia (time below 4 mmol/L) were also calculated.

Postprandial glucose control was defined as the iAUC above pre-meal glucose level (average of 30-min pre-meal) over the 2-h postprandial period for four individual meals (lunch, 15:00; dinner, 19:00; breakfast, 07:00; snack, 12:00). Total postprandial glucose iAUC was also calculated as the summation of iAUCs from all four meal periods. Such approach has been used frequently when assessing the effects of exercise on postprandial glucose outside of the laboratory (18, 20, 21). Post-absorptive insulin sensitivity was estimated using the homeostasis model assessment (HOMA-IR) (46). Whole-body substrate oxidation rates were calculated from V̇O_2_ and V̇CO_2_ at rest (47) and during exercise (48).

### Statistical Analysis

Either one- or two-way repeated-measures ANOVA were used where appropriate, and in the case of significant interaction effects, pairwise comparisons were made with Bonferroni corrections. Effect sizes for *post hoc* pairwise comparisons were calculated using Cohen’s d and are presented in the figure legends. All analyses were performed in GraphPad Prism 7.0 (La Jolla, CA) with a value of *P* < 0.05 considered to be statistically significant. Data are presented as mean ± SEM.

## Results

### Participant Characteristics and Pre-Trial Standardization

**Table 1** presents characteristics and habitual physical activity levels of participants recruited to complete the study. **Table 2** confirms adherence to pre-trial diet and activity standardizations, showing no between-trial differences in dietary intake, physical activity or CGM-derived glycemic control collected during 24-h pre-trial. There were also no significant changes in body composition (body mass, BMI and waist circumference) between trials (*P* > 0.05, data not shown).

**Table 2:** Physical activity, dietary intake and free-living CGM variables measured for 24 h before each experimental trial.

	Pre-trial standardizations			
	CON	NG-Ex	HG-Ex	FG-Ex
**Dietary intake**				
Energy intake, kcals	2250.3 ± 164.1	2116.3 ± 141.1	2043.8 ± 155.4	2080.5 ± 169.0
Carbohydrate intake (% of kcals)	47.0 ± 4.0	49.4 ± 4.6	47.0 ± 3.7	48.2 ± 4.3
Fat intake (% of kcals)	35.9 ± 2.0	33.5 ± 2.4	35.7 ± 2.1	34.7 ± 2.2
Protein intake (% of kcals)	17.1 ± 2.5	17.1 ± 2.4	17.3 ± 2.1	17.0 ± 2.4
**Physical activity**				
Step count, steps	10715 ± 1893	10722 ± 1725	10032 ± 1746	10229 ± 2048
Energy expenditure, kcals	587.9 ± 132.9	554.4 ± 115.1	529.1 ± 109.3	562.4 ± 122.3
Sedentary time, min	726.0 ± 67.6	619.1 ± 86.8	685.8 ± 72.4	753.1 ± 71.7
MVPA time, min	72.3 ± 17.2	70.8 ± 14.7	66.3 ± 13.9	68.9 ± 18.0
**CGM-derived variables**				
Mean 24 h glucose, mmol/L	5.2 ± 0.3	5.4 ± 0.1	5.5 ± 0.2	5.2 ± 0.1
Glucose variability, SD	0.9 ± 0.1	0.8 ± 0.1	0.9 ± 0.1	0.7 ± 0.1
Glucose variability, CV%	17.0 ± 1.4	14.7 ± 0.8	16.4 ± 1.7	13.7 ± 0.9

Data are mean ± SEM. No significant between-trial differences.

### Blood Biochemistry During the Experimental Trials

**Figure 2:**
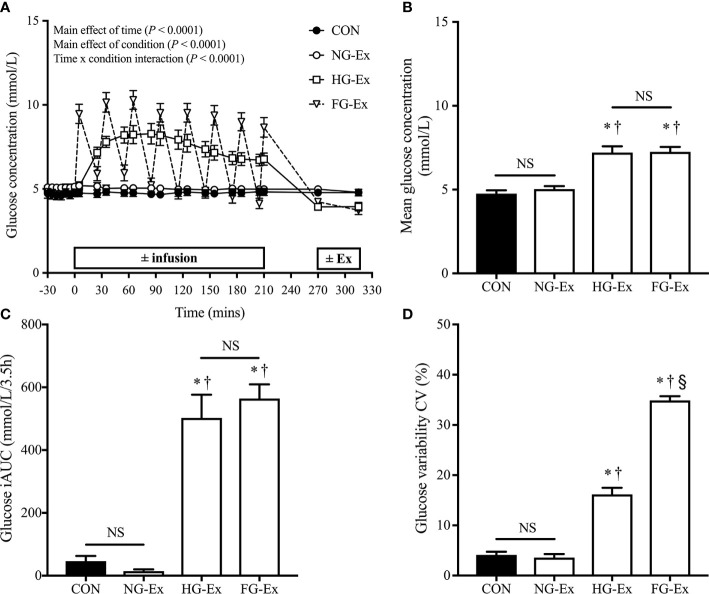
Glucose control during conditions of ± glucose infusion and ± exercise. **(A)** Time course for glucose concentration (mmol/L) during each experimental trial. **(B)** The mean glucose concentration (mmol/L) during each 3.5 h ± infusion period. *P < 0.05 (HG-Ex, *d* = 2.38; FG-Ex, *d* = 4.02) vs. CON. ^†^*P* < 0.05 (HG-Ex, *d* = 2.17; FG-Ex, *d* = 3.83) vs. NG-Ex. **(C)** Glucose iAUC during each 3.5 h ± infusion period. *P < 0.05 (HG-Ex, *d* = 2.47; FG-Ex, *d* = 4.40) vs. CON. ^†^*P* < 0.05 (HG-Ex, *d* = 2.70; FG-Ex, *d* = 4.94) vs. NG-Ex. **(D)** Glycemic variability (coefficient of variation; %CV) during each 3.5 h ± infusion period. *P < 0.05 (HG-Ex, *d* = 3.35; FG-Ex, *d* = 12.05) vs. CON. ^†^*P* < 0.05 (HG-Ex, *d* = 3.38; FG-Ex, *d* = 11.45) vs. NG-Ex. ^§^*P* < 0.05 (FG-Ex, *d* = 4.84) vs. HG-Ex.

**Figure 2A** shows that the glucose infusion protocol successfully induced three distinct glycemic profiles: normoglycemic (CON and NG-Ex) and two different hyperglycemic conditions (HG-Ex and FG-Ex). Mean glucose concentration (**Figure 2B**) and glucose iAUC (**Figure 2C**) during the 3.5 h ± infusion period were significantly higher during HG-Ex and FG-Ex compared to CON and NG-Ex (all comparisons *P* < 0.05), with no differences between CON and NG-Ex (*P* > 0.05). **Figure 2D** confirms that the FG-Ex trial had an unstable and fluctuating glucose profile, since CV%, a measure of glycemic variability, was significantly higher than in all other trials (all comparisons *P <* 0.05).

**Figure 3:**
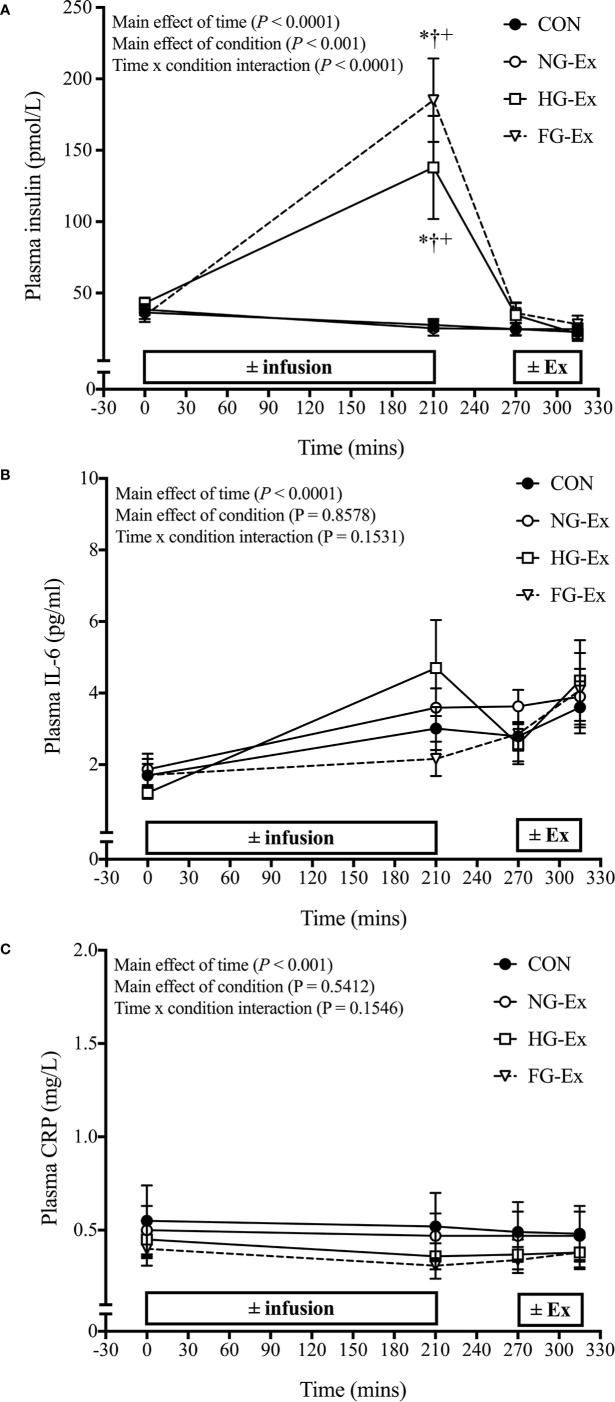
Time course for changes in plasma insulin, IL-6, and CRP. **(A)** Time course for plasma insulin (pmol/L) during each experimental trial. *P < 0.0001 (HG-Ex, *d* = 1.24; FG-Ex, *d* = 2.18) vs. time-matched CON; ^†^*P* < 0.0001 (HG-Ex, *d* = 1.26; FG-Ex, *d* = 2.20) vs. time-matched NG-Ex; ^+^*P* < 0.0001 (HG-Ex, *d* = 1.07; FG-Ex, *d* = 2.09) vs. pre-infusion. **(B)** Time course for IL-6 concentration (pg/mL) during each experimental trial. **(C)** Time course for CRP (mg/L) during each experimental trial.

**Figure 3A** shows that plasma insulin concentrations were significantly increased post-infusion vs. pre-infusion in both HG-Ex and FG-Ex (both *P* < 0.0001 vs. respective baseline), but not NG-Ex and CON (*P* > 0.05). Post-infusion values in HG-Ex and FG-Ex were also significantly higher than time-matched values in NG-Ex and CON (*P* < 0.0001 vs. time-matched values). There was no significant interaction effect of experimental treatments upon plasma IL-6 (**Figure 3B**) or plasma CRP (**Figure 3C**) concentrations (*P* > 0.05).

### Physiological Responses to Exercise During the Trials

**Figure 4:**
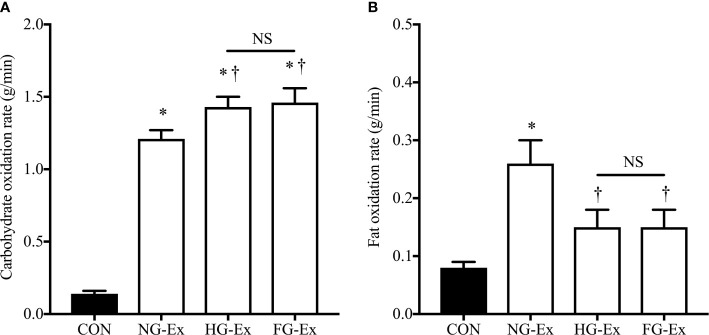
Rates of substrate oxidation during exercise. **(A)** Carbohydrate oxidation rates during the 45-min ± exercise period within the four experimental trials. *P < 0.05 (NG-Ex, *d* = 6.85; HG-Ex, *d* = 7.06; FG-Ex, *d* = 5.18) vs. CON. ^†^*P* < 0.05 (HG-Ex, *d* = 0.96; FG-Ex, *d* = 0.84) vs. NG-Ex. **(B)** Fat oxidation rates during the 45-min ± exercise period within the four experimental trials. *P < 0.05 (NG-Ex, *d* = 1.81) vs. CON. ^†^*P* < 0.05 (HG-Ex, *d* = 0.90; FG-Ex, *d* = 0.89) vs. NG-Ex.

The exercise/rest period lasted 44.6 ± 0.4 min and characteristics of these periods are shown in **Table 3**. Heart rate, power output, V̇O_2_, work done, and energy expenditure were similar between the three exercise trials (*P* > 0.05) and significantly higher than during the resting trial (*P* < 0.05). RER was significantly higher during the three exercise trials vs. CON (*P* < 0.05; **Table 3**), and also significantly higher in HG-Ex and FG-Ex compared to NG-Ex (*P* < 0.05; **Table 3**). Furthermore, carbohydrate oxidation rates during exercise/rest were significantly higher in all three exercise trials (NG-Ex, HG-Ex, and FG-Ex) compared to CON (*P* < 0.05), with rates during HG-Ex and FG-Ex also significantly higher than NG-Ex (*P* < 0.05; **Figure 4A**). Fat oxidation rates during exercise in NG-Ex were significantly higher than all other trials (*P* < 0.05), with no further between-condition differences (*P* > 0.05; **Figure 4B**).

**Table 3:** Physiological responses to acute exercise intervention.

	CON	NG-Ex	HG-Ex	FG-Ex
Duration, min	44.6 ± 0.4	44.6 ± 0.4	44.6 ± 0.4	44.6 ± 0.4
Heart rate, bpm	61 ± 2	132 ± 2*	131 ± 2*	130 ± 2*
Heart rate, %HRmax	32.2 ± 1.2	69.5 ± 0.4*	68.7 ± 0.7*	68.1 ± 0.7*
Power output, W	0.0 ± 0.0	88.6 ± 4.8*	88.0 ± 5.1*	90.3 ± 4.7*
Power output, %Wmax	0.0 ± 0.0	33.1 ± 0.7*	32.9 ± 1.3*	33.9 ± 1.2*
V̇O_2_, ml/min/kg	4.0 ± 0.3	22.0 ± 1.6*	21.5 ± 1.2*	21.5 ± 1.1*
V̇O_2_, %V̇O_2_max	10.0 ± 0.5	55.0 ± 3.5*	53.3 ± 2.4*	54.7 ± 4.0*
Work done, J	0.0 ± 0.0	3884.7 ± 209.2*	3875.3 ± 223.3*	3950.7 ± 214.6*
Energy expenditure, kcals	61.7 ± 3.6	326.1 ± 18.8*	322.9 ± 15.0*	324.0 ± 14.6*
RER, a.u.	0.82 ± 0.01	0.90 ± 0.01*	0.94 ± 0.01*†	0.94 ± 0.01*†

Data are mean ± SEM. *P < 0.05 vs. CON. †P < 0.05 vs. NG-Ex.

### Post-Trial Standardization

**Table 4** confirms that both dietary intake and physical activity levels during the 24-h post-trial period were similar between trials (*P* > 0.05).

**Table 4:** Physical activity, dietary intake, and free-living CGM variables measured for 24 h following each experimental trial.

	Post-trial standardizations			
	CON	NG-Ex	HG-Ex	FG-Ex
**Dietary intake**				
Energy intake, kcals	2467.6 ± 4.6	2467.6 ± 4.6	2467.6 ± 4.6	2467.6 ± 4.6
Carbohydrate intake (% of kcals)	54.9 ± 0.1	54.9 ± 0.1	54.9 ± 0.1	54.9 ± 0.1
Fat intake (% of kcals)	31.6 ± 0.1	31.6 ± 0.1	31.6 ± 0.1	31.6 ± 0.1
Protein intake (% of kcals)	13.5 ± 0.1	13.5 ± 0.1	13.5 ± 0.1	13.5 ± 0.1
**Physical activity**				
Step count, steps	8729 ± 1333	8895 ± 853	7355 ± 998	8661 ± 1260
Energy expenditure, kcals	476.4 ± 63.8	474.7 ± 50.1	397.3 ± 60.0	473.6 ± 76.9
Sedentary time, min	711.5 ± 75.3	733.8 ± 66.7	764.7 ± 60.7	709.5 ± 64.5
MVPA time, min	56.8 ± 10.4	55.2 ± 6.5	42.5 ± 7.7	55.6 ± 10.9
**CGM-derived variables**				
Mean 24-h glucose, mmol/L	5.3 ± 0.2	5.2 ± 0.1	5.3 ± 0.2	5.3 ± 0.2
Glucose variability, SD	0.8 ± 0.1	0.7 ± 0.1	0.7 ± 0.1	0.7 ± 0.1
Glucose variability, CV%	14.1 ± 0.8	14.1 ± 1.1	13.8 ± 0.9	14.3 ± 1.3

Data are mean ± SEM. No significant differences.

### Post-Trial 24-h Glycemic Control

There were no significant between-condition differences in 24-h glucose exposure (i.e., mean 24-h glucose concentrations) during the post-exercise period (*P* > 0.05; **Table 4**). Since the regulatory mechanisms (e.g., changes in glucose uptake and insulin sensitivity) of exercise-induced glucoregulatory benefits follow time-dependent profiles (15), the 24-h post-exercise period was broken down to discreet periods, namely: 0–6, 0–12, 0–18, 6–12, 6–18, 6–24, 12–18, 12–24, and 18–24 h. However, there were no significant between-condition differences in mean glucose during these periods (*P* > 0.05). Measures of 24-h glycemic variability, namely, SD and %CV, were not significantly affected by the experimental conditions (**Table 4**, both *P* > 0.05). Similarly, there were no significant between-condition differences in the 24-h prevalence of hyperglycemia or hypoglycemia (both *P* > 0.05; data not shown).

### Post-Trial Postprandial Glycemic Control

**Figure 5:**
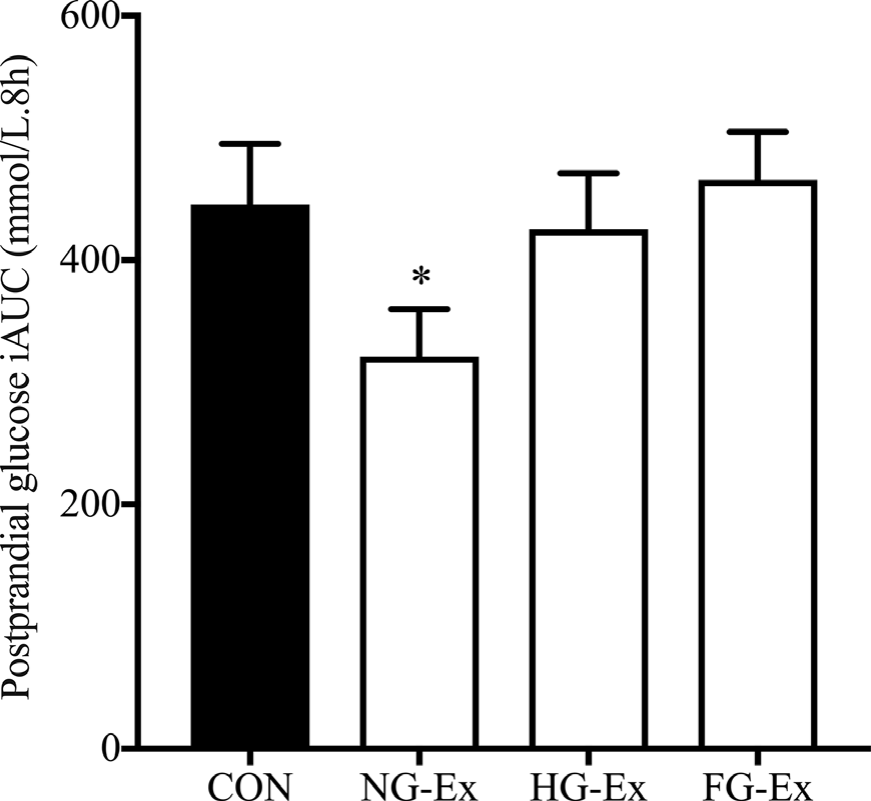
The effects of the four interventions on total postprandial glucose iAUC. Total postprandial glucose response (the sum of post-meal glucose iAUC across four standardized meals) was measured using CGM following four experimental trials. *P < 0.05 (NG-Ex, *d* = 0.81) vs. CON.

There were no significant between-condition differences in mean glucose or glucose iAUC in the 2-h postprandial period following the four meals provided when analyzed per meal (*P* > 0.05). However, as presented in **Figure 5**, total postprandial glucose response, measured as the sum of post-meal glucose iAUC, was significantly reduced by exercise in NG-Ex compared to CON (321.1 ± 38.6 mmol/L.8h vs. 445.5 ± 49.7 mmol/L.8h, *P* < 0.05). However, such benefits were blunted when exercise was preceded by steady-state hyperglycemia (425.3 ± 45.7 mmol/L.8h) and fluctuating glycemia (465.5 ± 39.3 mmol/L.8h) in HG-Ex and FG-Ex, respectively (both *P* > 0.05 vs. CON).

## Discussion

The current study is the first to experimentally investigate the direct effect of hyperglycemia on exercise-induced benefits in glucose control. The findings demonstrate that pre-exercise hyperglycemia blunts the exercise-induced improvement in postprandial glucose response following a single exercise bout in healthy normal glucose-tolerant individuals.

Excessive postprandial glucose exposure contributes to the worsening of HbA1c in non-diabetic individuals (7), as well as exacerbating the risk of developing diabetic complications and premature mortality (4, 6). Accordingly, reducing postprandial glucose is a prime therapeutic target in the prevention and management of hyperglycemia-related conditions, such as T2DM. In this study, when exercise was preceded by normoglycemia, total postprandial glucose iAUC was significantly reduced compared to the non-exercise CON condition (*d* = 0.81; **Figure 5**). While reference ranges for CGM-derived treatment targets are constantly being refined (45, 49), a reference range for what constitutes a clinically important exercise-induced change in a CGM-derived variable does not currently exist. Nevertheless, the improved postprandial glucose response in the current study is in agreement with previous research also demonstrating the potency of exercise in improving this specific CGM-derived measure of glycemic control (18, 20, 21). However, this beneficial effect was blunted when the same individuals were exposed to steady-state hyperglycemia or fluctuating glycemia before exercise (**Figure 5**). This is the first experimental evidence demonstrating a direct effect of pre-exercise hyperglycemia upon the potency of exercise to induce glucoregulatory benefits. The findings, therefore, support that hyperglycemia contributes to the inter-individual heterogeneity of the metabolic response to exercise.

The magnitude and profile of an individual’s postprandial glucose response are determined by multiple factors. These include meal characteristics such as caloric content and macronutrient composition, but also the nutrient-induced gastrointestinal responses, tissue glucose uptake, insulin sensitivity, pancreatic β-cell insulin secretory function, glucose effectiveness, and hepatic and renal glucose handling (50–53), all of which are regulated by exercise. Therefore, the exercise-induced enhancement in postprandial glucose control seen in the NG-Ex trial is likely attributable to coordinated exercise-mediated enhancements in any of the above factors. As a corollary, if pre-exercise hyperglycemia impairs any of these adaptive processes, this would feasibly blunt exercise-induced improvements in postprandial glucose response. This notion is supported by evidence demonstrating that chronic hyperglycemia is associated with smaller exercise-induced improvements in peripheral insulin sensitivity, which in turn was linked with blunted exercise-induced improvements in postprandial glucose response (54).

Furthermore, hyperglycemia impairs skeletal muscle cell insulin sensitivity (55–58), pancreatic β-cell insulin secretory capacity (59, 60), and endothelial cell function (61, 62) within *in vitro* experimental models. Likewise, skeletal muscle (63, 64) and hepatic (65) insulin sensitivity, insulin secretory capacity (63), and vascular function (66) are impaired by exposure to experimental hyperglycemia *in vivo*, even in healthy individuals with normal glucose tolerance. Such hyperglycemia-induced impairments could also explain the elevated postprandial glucose response following the HG-Ex and FG-Ex trials.

Impaired function of key glucoregulatory tissues, including muscle, liver, and the endocrine pancreas, may also contribute indirectly to poorer postprandial glucose response following HG-Ex and FG-Ex since the impaired function of these tissues at baseline has also been associated with poorer outcomes following exercise. For example, blunted exercise-induced improvements in insulin sensitivity have been documented in individuals with higher baseline insulin resistance (54), and poorer contraction-induced improvements in glucose metabolism and insulin sensitivity has been found in primary myotubes from insulin resistant (e.g., obese and/or T2DM) vs. healthy donors (67, 68). Similarly, poorer baseline pancreatic β-cell function is predictive of poorer exercise responsiveness (33). Collectively, in this study, pre-exercise hyperglycemia may have impaired the function of glucoregulatory tissues (muscle, pancreas, etc.) while also blunting their ability to respond to exercise.

Markers of inflammation and oxidative stress are elevated in T2DM and are increased by experimental hyperglycemia (66). Since excessive levels of inflammation and oxidative stress can impair exercise adaptations (69), pre-exercise hyperglycemia-induced inflammation and oxidative stress may influence exercise-induced benefits. However, we found no significant interaction effect in two markers of systemic inflammation (IL-6 and CRP; **Figure 3**) suggesting that inflammation may not explain our findings. That said, tissue inflammation was not examined and these outcomes may be different in a patient population. Alternatively, pre-exercise hyperglycemia appeared to promote carbohydrate utilization (**Figure 4A**) and suppress fat utilization (**Figure 4B**) during exercise compared to exercise under normoglycemic conditions despite matched exercise stimuli (**Table 3**). Such alterations in substrate use during exercise may contribute to altered glucoregulation during the post-exercise period.

In contrast to the exercise-induced improvements in postprandial glucose, there were no significant effects of exercise upon mean 24-h glucose concentration, glucose SD or glucose CV%. This contradicts the improvements in 24-h glycemic control seen in hyperglycemic participants (20, 38, 40, 70). The smaller margin for improvement in the normal glucose-tolerant participants in the current study (HbA1c = 5.4 ± 0.1%) compared to the aforementioned studies in hyperglycemic individuals may contribute to this discrepancy. Interestingly, there were also no significant effects of HG-Ex or FG-Ex on these markers of 24-h glycemic control compared to either normoglycemic condition, highlighting that the ability of healthy humans to regulate glucose homeostasis under a challenge from distinctly different stimuli (i.e., exercise and hyperglycemia) is remarkably well-preserved.

Given the aim of the current study to investigate possible contributing factors to response heterogeneity in glycemic improvements following exercise, it would be remiss to not discuss variability in outcomes. Inter-individual heterogeneity showed that 9, 7, and 7 out of 12 individuals showed a decrease in 24-h mean glucose, glucose SD, and glucose CV%, respectively, following exercise. This variability in responses likely contributes to the lack of significance seen for these outcomes. A further anecdotal observation is that pre-exercise hyperglycemia in HG-Ex and FG-Ex reduced the participants’ enjoyment during exercise when compared to NG-Ex. This is important because reduced enjoyment during exercise could feasibly impact exercise adherence in a free-living setting.

In the present study, healthy, recreationally active, non-diabetic individuals were exposed to blood glucose profiles similar to those seen in T2DM (i.e., elevated and unstable) for 3.5 h. It is important to note that the short-term, acute nature of this likely represents a different metabolic and physiological challenge compared to the chronic state of hyperglycemia and/or glycemic instability in T2DM. Nevertheless, the approach used enabled us to isolate the effect of acute hyperglycemia from other symptoms or comorbidities of T2DM (e.g., chronic low-grade inflammation, dyslipidaemia, impaired insulin sensitivity and/or secretion, etc.), while also avoiding changes in circulating incretin hormones (e.g., GLP-1) otherwise induced by oral glucose ingestion (71), thus justifying the study design used. That said, since pancreatic clamp conditions were not employed during glucose infusion in the current study, the possibility that increased plasma insulin in hyperglycemic (HG-Ex and FG-Ex) vs. normoglycemic (NG-Ex and CON) conditions (**Figure 3A**) contributed to the responses seen cannot be excluded. Additionally, short-term hyperglycemia, such as during a 2-h hyperglycemic clamp, reduces plasma glucagon concentration, and suppresses endogenous glucose production in healthy individuals (63), and such effects may contribute to responses observed in the current study. Furthermore, since exercise in the current study is acute and of moderate-intensity, whether exercise within training regimes and/or of greater intensity elicits the same outcomes remains to be determined. Future studies should also aim to quantify the long-term impact of changes in CGM-derived outcomes on the risk of developing hyperglycemia-related conditions/complications.

Using glucose infusion to induce hyperglycemia introduced caloric and carbohydrate imbalances between trials. Specifically, there was an energy deficit in CON (−61.7 ± 3.6 kcals) and NG-Ex (−326.1 ± 18.8 kcals) and a slight energy surplus in HG-Ex (+10.9 ± 20.5 kcals) and FG-Ex (+9.8 ± 17.7 kcals). Similarly, while more carbohydrate was oxidized during exercise within the infusion trials compared to NG-Ex (**Figure 4A**), this difference does not account for the amount of glucose infused during HG-Ex and FG-Ex. While the thermic effect of glucose infusion itself, which increases resting energy expenditure (by ~5%–11%) (72–74) and carbohydrate oxidation (75), may attenuate these between-trial imbalances to some extent, the possibility that they contributed to findings cannot be excluded. Excess glucose may have increased liver (76) and skeletal muscle (64) glycogen storage which may impact the capacity for postprandial glucose disposal during the post-exercise period in the infusion trials only. That said, while glucose infusion to maintain glucose concentrations at +2.5 mmol/L above fasting for 3 days doubled muscle glycogen (64), the short-term, acute nature of infusion in the current study (3.5 h) is unlikely to induce such a stark increase. Additionally, moderate-intensity exercise still reduces muscle glycogen under conditions of experimental hyperglycemia (via glucose infusion) (77) and when pre-exercise muscle glycogen is elevated (78). Furthermore, post-exercise glycogen storage is not solely reliant on changes at the muscle itself, but also splanchnic bed responses that increase the rate of oral glucose appearance in the circulation (79).

Compensating for the aforementioned imbalances with altered food provision without several additional control trials would introduce other confounding variables (e.g., differences in macronutrient provision), meaning that imbalance of some form is somewhat inevitable. Therefore, in line with many studies evaluating the effects of exercise vs. rest (20, 21, 38) or normo- vs. hyperglycemia (63, 64) upon glucose metabolism, compensation of these caloric and carbohydrate imbalances were not included, with the meals kept constant across all conditions. Moreover, this should not detract from the significance of the findings since the exercise-induced improvements in the function of key glucoregulatory tissues (e.g., muscle, pancreas, liver, gastrointestinal tract, etc.) that contribute to improved postprandial glucose result from more than simply an energy and glycogen deficit.

A key methodological strength of the current study is the balance between having strict control of pre-trial and post-trial standardization, glycemic interventions, exercise interventions, and dietary control, while monitoring clinically relevant outcomes (i.e., CGM-derived glycemic control) in an otherwise free-living, ecologically-valid setting. Additionally, unlike some previous exercise vs. control studies using CGM-derived outcomes (18, 70, 80), trial order was randomized to minimise the risk of bias. The consumption of the same controlled diet and the same physical activity levels across all 24-h post-trial periods increases the confidence that observed effects upon postprandial glucose are likely attributable to differences in the experimental treatments (i.e., ± glucose infusion, ± exercise) during the respective trials.

In the context of ecological validity, this study implicates a direct inhibitory effect of pre-exercise hyperglycemia upon exercise-induced improvements in postprandial glucose control. Therefore, it could be speculated that reducing hyperglycemia before exercise and/or coinciding exercise sessions with periods of improved glucose control (i.e., lower, more stable levels) may be a necessary strategy for optimizing the therapeutic effects of exercise in individuals with hyperglycemia-related conditions, such as T2DM. Hyperglycemia-lowering medication, dietary interventions, or optimizing exercise-meal timing may provide viable options in this regard—that said, both diet and medication have themselves been implicated in variable exercise responsiveness (27, 30). Therefore, an individualized approach to glucose management will likely be best and more work in that area is required. Future work must also resolve how the findings translate to longer-term exercise training interventions in patients with T2DM before extrapolation to disease management. That said, large portions of the worldwide population are non-diabetic meaning these results hold clinical significance in the context of disease prevention.

In conclusion, this study provides the first experimental evidence that pre-exercise hyperglycemia can blunt the glucoregulatory benefits of acute exercise, suggesting that hyperglycemia contributes to exercise response heterogeneity. Resolving hyperglycemia before exercise and/or coinciding exercise sessions with periods of improved glucose control may, therefore, be vital for maximizing the therapeutic effects of exercise in individuals with and at risk of hyperglycemia-related conditions, such as T2DM.

## Data Availability Statement

The raw data supporting the conclusions of this article will be made available by the authors, without undue reservation.

## Ethics Statement

Ethical approval was obtained through the West Midlands - South Birmingham Research Ethics Committee (16/WM/0242) and sponsored by the University of Birmingham Research Governance. All participants provided written informed consent before participation.

## Author Contributions

SC and TPJS conceived the idea, designed the study, and implemented the trials. SC completed the data analysis and wrote the manuscript. SC and TPJS interpreted the findings and discussed the data. TPJS edited the manuscript. All authors agree to be accountable for the content of the work. All authors contributed to the article and approved the submitted version.

## Funding

The study was funded in part by the Physiological Society and the European Commission (Marie Skłodowska-Curie Individual Fellowship).

## Conflict of Interest

The authors declare that the research was conducted in the absence of any commercial or financial relationships that could be construed as a potential conflict of interest.
