# Supplementary figures and images for: Exercise-Induced Improvements in Postprandial Glucose Response Are Blunted by Pre-Exercise Hyperglycemia: A Randomized Crossover Trial in Healthy Individuals
Source: Front Endocrinol (Lausanne). 2020 Oct 15;11:566548. doi: 10.3389/fendo.2020.566548 (PMC7593662; doi:10.3389/fendo.2020.566548)

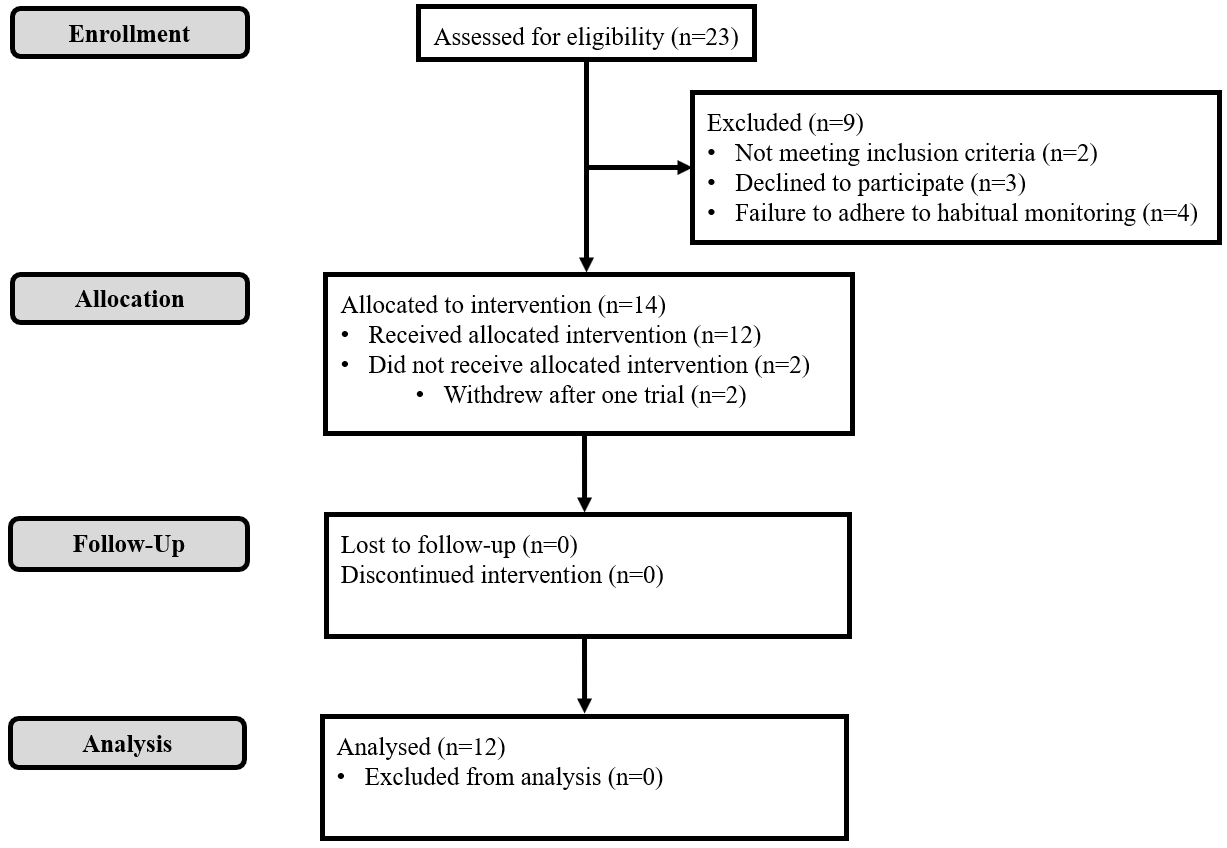

Supplement: Supplementary file 1 [file Image_1.tif]
